# Kinetic description of changes in the size of casein microparticles under simulated gastric conditions

**DOI:** 10.1038/s41598-025-22216-7

**Published:** 2025-10-10

**Authors:** Ronald Gebhardt, Calvin Hohn

**Affiliations:** Chair of Soft Matter Process Engineering (AVT.SMP), RWTH Aachen University, 52074 Aachen, Germany

**Keywords:** Biophysics, Computational biology and bioinformatics

## Abstract

Casein microparticles, which are gently produced by depletion flocculation and film drying, are suitable as a sustainable, biodegradable encapsulation material due to their pH-dependent swelling behaviour. However, the food protein casein is highly susceptible to degradation in the stomach due to its open, flexible conformation. In simulated gastric juice, the particles undergo different phases of shrinkage and swelling. Control experiments show that the initial two-stage shrinkage process and the subsequent swelling are due to the acidification of the particles. When pH 2 is reached, an almost exponential decay induced by pepsin takes place until a highly degraded final state is reached. The kinetic analysis of the results is based on the pH profile of the casein solubility as well as on pH- and pepsin-induced structural changes in the casein matrix, which are combined in a system-dynamic model. Data and model simulations show that microparticles can pass through the gastric environment.

**Supplementary Information:**

The online version contains supplementary material available at 10.1038/s41598-025-22216-7.

## Introduction

The use of casein as an encapsulation material for bioactive substances and pharmaceuticals has recently attracted considerable research interest^1–3^. Caseins can be easily extracted from cow’s milk and, as natural biopolymers, are a sustainable and biodegradable alternative to petroleum-based polymers^4^. Under natural conditions, a total of four different casein molecules (α_S1_-, α_S2_-, β- and κ-casein with contents of 40, 11, 37 and 12%) are organized together with minerals as association colloids^5^, historically called casein micelles^6^. One physiological function of casein micelles is to provide newborns with sparingly soluble calcium phosphate via milk. The mineral is present in the form of colloidal calcium phosphate nanoclusters and is linked via calcium bridges to the phosphoserine clusters of the calcium-sensitive caseins inside the micelle^7–9^.

In addition to the natural carrier function for calcium phosphate, micellar casein can also be functionalized for the transit of bioactive substances through the gastrointestinal tract^10–12^. In particular, due to their size, casein microparticles are considered to be protective capsules for microorganisms against gastric and intestinal fluids^13,14^. Apart from microorganisms, small bioactive molecules have also been successfully encapsulated, including the peptide hormone insulin^15^ and the polyphenol quercetin^16^. These examples demonstrate the broad range of applications of casein microparticles as a carrier system.

**Fig. 1:**
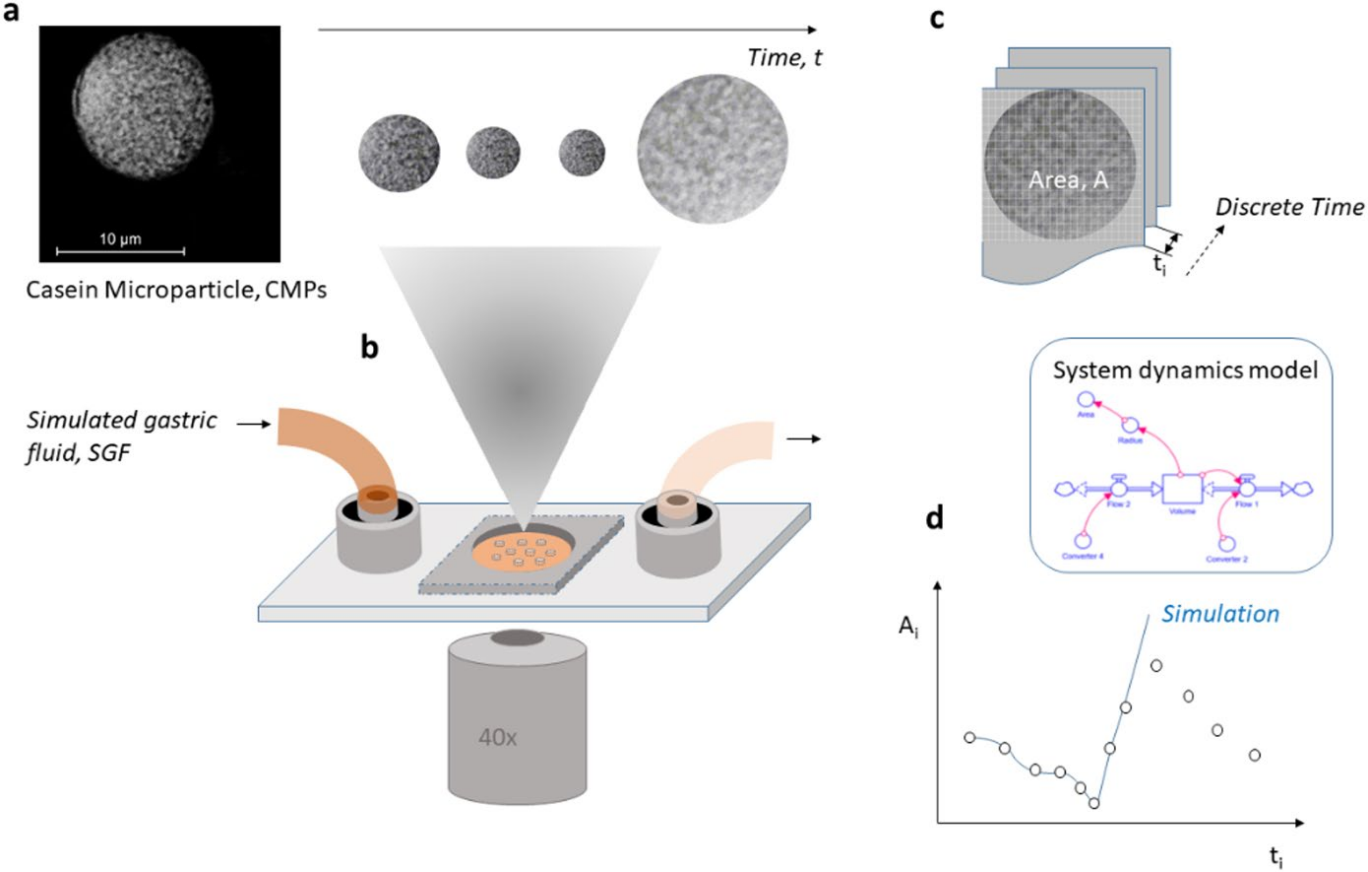
Overview of the main steps of the experimental approach and analysis: **a**) Several µm-sized, spherical CMPs with a sponge-like internal structure; **b**) Size changes of individual CMPs, observed microscopically in the sieve holes of a flow cell, after overflow, e.g. with simulated gastric fluid, SGF; **c**) Determination of the projection area of the CMPs at discrete time points of the kinetics; **d**) System-dynamic modelling and simulation of the complex structural changes.

Many substances are sensitive to the conditions in which they are encapsulated, such as high mechanical stress, pressure, temperature or pH extremes. We have developed a process to gently produce casein microparticles (CMPs) from casein micelles at neutral pH and without the use of harmful chemicals under ambient temperature and pressure conditions^17^. The CMPs are spherical with sizes between 10 and 50 μm and have a sponge-like, open-pored internal structure (Fig. 1a). Their characteristic swelling behavior can be studied on individual particles. To do this, they are placed in the sieve holes of 3D printed flow-through cells while the medium is exchanged (see Fig. 1b)^18^. The structural changes are recorded microscopically and the projection areas of the particles are determined at discrete time points (Fig. 1c). Due to the open, flexible conformation of the caseins and the fact that the spherical shape of the CMPs is maintained until disintegration, the changes in the projection areas can be attributed to volume flows over the particle surface and modelled using a system dynamics approach (Fig. 1d).

CMPs are a promising encapsulation material for gastrointestinal transit due to their food-safe and gentle manufacturing conditions and pH-dependent stability. However, after oral ingestion, caseins and casein capsules are exposed to the harsh pH conditions and proteases of the stomach. For adults, the gastric fluid has a pH value between 1 and 2, but this can initially rise to over pH 6 after ingestion due to low buffering capacity. In the course of digestion, the pH value then falls almost linearly within 60 min^19^, although the rate varies depending on the protein ingredients^20^. In dairy products made from casein micelles, the enzymatic destabilization of the κ-casein surface layer^21^ leads to the formation of a gastric coagulum right at the start of gastric digestion, which is amplified by a further drop in pH^22^. Since pepsin has a maximum activity at pH 2, a strong pH dependence of enzymatic degradation is observed at the beginning of the digestive process, which lasts several hours^23^. The structure of the coagulum is decisive for the further course of digestion, which depends largely on the structure of the clots, the content of colloidal calcium and protease activity^24,25^.

While the milk protein alone and the binding of bioactive compounds to it have been very well studied, there is little understanding of the behavior of caseins as wall material of capsules and methods to investigate the influence of relevant environmental and process conditions on it^26^. In their study, Liao et al. highlight the importance of the wall material for drug release and the need for further research^27^. It is also important to mention that regulated enzymatic degradation and controlled swelling of the microparticle wall material are two relevant strategies for the controlled release of bioactive substances^28^. The swelling also influences the rate of gastric digestion, as shown in protein gels^29^.

Since CMPs retain their spherical shape during swelling and shrinkage until shortly before disintegration, size changes can be analyzed microscopically and simulated using a system dynamics approach based on an underlying model in the form of a system of differential equations. In this in vitro study, we analyze the structural changes of CMPs during simulated gastric digestion. We observe size changes of individual microparticles microscopically in a flow cell while they are overflown by SGF with gastric-like pH values and added pepsin. Using control experiments, we distinguish between pH- and enzyme-induced effects and analyze the observed size changes. Based on individual kinetic analyses, we are developing a system dynamics model that provides a platform for future coupling of structural changes with release kinetics.

## Results and discussion

**Fig. 2:**
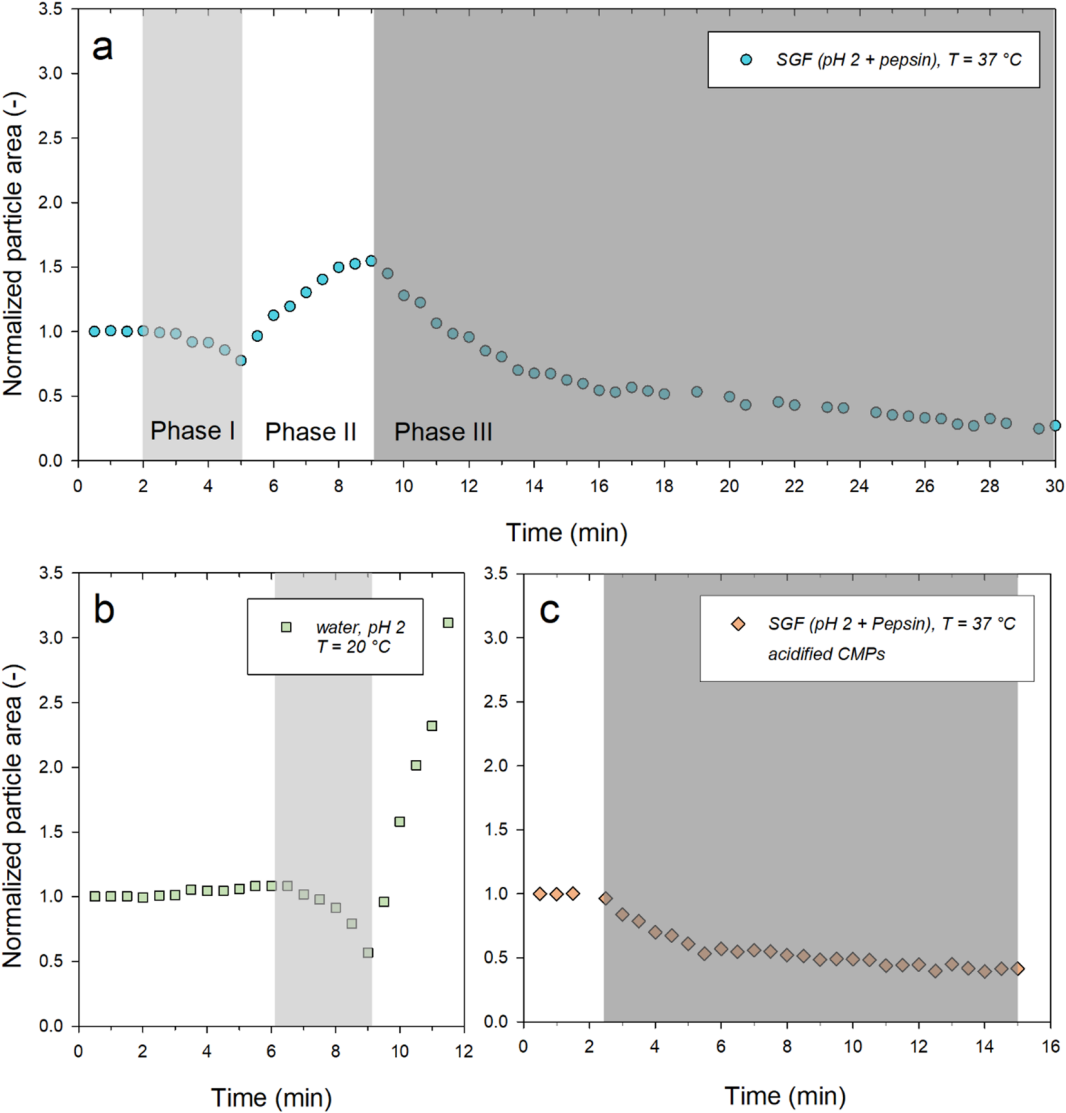
Relative size change of individual representative CMPs as a function of time. **a**) After pH change from neutral to pH 2 with SGF, with addition of 800 u pepsin/ml; **b**) After change to pH 2 using ultrapure water and **c**) After addition of SGF with 50 u pepsin/ml and prior storage in SGF (pH 2) for 20 min. The periods when similar shrinkage was observed are highlighted in the same grey colors.

The effect of a pH reduction and pepsin activity on the size of CMPs was investigated at T = 37 °C to better understand the influence of the gastric environment on the stability and swelling behavior of the particles. At the start of the experiment, the sieve of an optically transparent flow cell (Fig. 1b) was loaded with CMPs dispersed in neutral pH buffer. The medium was then replaced by SGF adjusted to pH 2 and containing pepsin. The resulting changes in the particle area were recorded microscopically. Two control experiments were also carried out. The CMPs were exposed to an aqueous medium with pH 2 at 20 °C, but without pepsin. In a further experiment, the influence of pepsin was tested at T = 37 °C on CMPs that had previously been acidified with SGF for 20 min at the same temperature. For all three experimental conditions, representative kinetics were selected from a series of single particle experiments and plotted in Fig. 2.

Figure 2a shows the change in particle area over time after the pH value of the surrounding medium had dropped to pH 2 due to exchange with SGF in the sieve cell. From the 2nd minute onwards, the particle shrinks in two stages within 3 min to approx. three quarters of the initial area (time range marked in light grey). From the 5th minute onwards, abrupt swelling occurred. The particle area initially expands very fast and later at a slower rate. The expansion does not end as usual in particle decay, but is followed from the 9th minute by exponential deswelling to a constant final value (area marked in dark grey).

If CMPs are acidified to pH 2 with ultrapure water instead of SGF, a slight initial swelling occurs (Fig. 2b). From the 6th minute onwards, a two-step deswelling also takes place, which is followed after approx. 3 min by a two-step expansion process. As already observed in Fig. 2a, this initially starts at a high rate and then slows down until the particle dissolves. As this control experiment did not contain any enzymes, the measurement was carried out under standard conditions at room temperature (20 °C). However, comparing this with a measurement carried out at 37 °C shows that temperature has no significant influence on the characteristic curve (see Fig. S1, supplementary materials). This control experiment using aqueous exchange medium without pepsin proves that the initial deswelling and subsequent swelling are due to acidification of the CMPs and not to enzyme activity.

In contrast, the final exponential decomposition process in Fig. 2a can be attributed to pepsin activity. If CMPs are first equilibrated for 20 min in SGF without pepsin at a pH of 2, they do not decompose as in ultra-pure water (see Fig. 1b), but probably remain stable due to the calcium ion content of SGF^30^. Only the subsequent exchange of the medium with SGF, pH 2 with added pepsin and an activity of at least 50 u/mL leads to an almost exponential decay to a constant plateau value, as shown in Fig. 2c.

The analysis of the above-mentioned changes in size of CMPs is based on the assumption that the rate of volume change during the swelling and shrinking process is only due to volume flows in and out of the particle according to the following formula:

1 $$\:\frac{dV}{dt}={\sum_{i}}{{I}_{Vi}}$$

In contrast, we neglect possible sources and sinks of volume in the balance, which could occur, for example, due to changes in the water binding to the casein. Because of the natively unfolded conformation of the caseins^31^ and the fact that the majority of the water is trapped and not bound within the colloidal state^32^, we estimate such contributions to be comparatively small. The volume flows for the observed simple swelling and shrinking processes

2 $$\:{I}_{V}=\pm\:V\cdot\:{\sigma\:}^{{t}^{*}}\cdot\:RC$$

depend on the current volume V and a rate coefficient RC^33^. The coefficient is activated from a characteristic time t* via a step function, σ, according to

3 $$\:{\sigma\:}^{{t}^{*}}=\left\{\begin{array}{c}0\:\:if\:t<{t}^{*}\\\:1\:\:if\:t\ge\:\:{t}^{*}\end{array}\right.$$

As the spherical shape of the CMPs is maintained throughout the swelling and shrinking process, the microscopically measured circular area can be recalculated using the spherical approximation

4 $$\:A=\frac{3\cdot\:V}{4\cdot\:R}$$

from the volume obtained by integrating Eq. 1 and adjusting to the measured data.

### Analysis of the initial shrinkage process (Phase I)

Figure 2a shows that CMPs shrink when exposed to SGF at pH 2. However, the fact that particle area values fall below the original size is also observed when CMPs are exposed to water at pH 2 (Fig. 2b). This directly indicates that the reduction in particle area is due to acidification and not to pepsin activity. Shrinking of the CMPs has so far only occurred during a sequence of cyclic pH changes between pH 11 and pH 1. However, after the initial swelling in the alkaline medium, the CMPs in the acidic medium only shrank to the original size they had at the beginning of the experiment under neutral pH conditions^33^.

According to Eq. 1, the reduction in particle size is caused by an outflow of solvent, which is squeezed out of the gel network of the microparticles. To understand the mechanism of this volume reduction during acidification to pH 2, it is necessary to consider the protein-protein and protein-solvent interactions and structural features of the casein micelle at the relevant pH range. These include the micellar bound calcium phosphate on the one hand and the hydration of the κ-casein surface layer on the other. The latter gradually collapses during acidification^34^. As a result, the attractive interactions between the casein micelles increase and the hydration of the micelle decreases^32^. The organic (bound) and inorganic phosphate within the micelles also increasingly loses ionisation due to proton binding, while the calcium and phosphate content in the aqueous phase increases due to mineral dissociation^35^. Despite the drastic changes at the level of caseins, water and minerals, the integrity of the casein micelle is maintained during acidification^34^.

Under neutral conditions at the beginning of the experiment, caseins are negatively charged. During acidification, phosphoseryl− and carboxyl−groups in particular bind more and more protons until the charges are neutralized at the isoelectric point at about pH 4.2. Like many other proteins, caseins have a minimum solubility in the pH range close to their isoelectric point^36^.

**Fig. 3:**
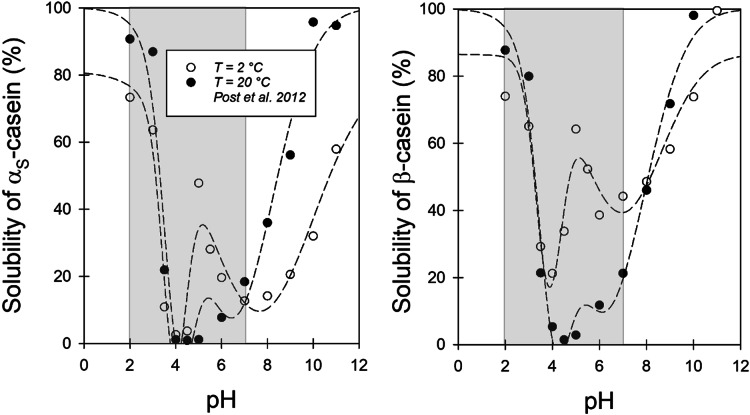
Analysis of pH-dependent solubility for α_S_-casein (left) and β-casein (right) with Eq. 5 (data taken from Post et al. 2012^37^.

In previous study, Post et al. investigated the pH dependence of the solubility of the two main proteins α_S_- and β-casein in micellar casein solution at T = 2 °C and 20 °C. As shown in Fig. 3, the solubility of both caseins passes through two minima in the range between pH 2 and 7, with a local maximum in between. The solubility around the maximum is significantly increased for both caseins at T = 2 °C, which can be attributed to weaker hydrophobic interactions between the proteins in the cold^37^. Since the casein microparticles investigated in this work contain all four main casein fractions and α_S_- and β- casein make up the majority of the total protein, their solubility behavior can be considered representative of the entire system. Consequently, the data can be utilized to analyze the interactions between the caseins, as well as between the caseins and the solvent within the matrix of the CMPs, which can be presumed to resemble casein micelles.

The pH range of interest for the acidification process studied here is highlighted in grey in Fig. 3. At 20 °C, the solubility of both α_S_ − and β−casein initially drops to the first minimum value at pH 6, as protonation reduces the electrostatic repulsion between the caseins and increases the interaction between the polymer chains. However, calcium phosphate contacts also dissociate, increasing the solubility of the caseins again and leading to the local solubility maximum in the region of pH 5.3^38,39^. In the cold, the binding of colloidal calcium phosphate to β-casein is significantly reduced compared to 20 °C, which, in addition to the weaker hydrophobic contacts, explains the higher solubilities at 2 °C in this range^37,40^. In the pH 5–4.7 range, the κ-casein layer collapses at the surface of the casein micelles^34^. The loss of the steric repulsion barrier in turn increases the aggregation tendency and decreases the solubility. The solubility minimum at pH 4 is finally close to the isoelectric point, where the repulsive forces between the caseins are most reduced and the aggregation tendency is maximum. If the pH is lowered further to pH 2, the solubility increases again, as the caseins become positively charged and the electrostatic repulsion between the polymer chains increases.

It seems plausible that the degree of insolubility determines the strength of the volume outflow during the shrinkage process. The greater the insolubility of the caseins, the more trapped water can be squeezed out of the gel network via the processes described above. In order to integrate this approach into the kinetic description, a simple functional relationship was identified for the solubility data of Post et al. in Fig. 3. All pH curves with the two minima can be described in good approximation by two density functions p1 and p2 according to:

5 $$\:{S}_{x-cas}\left(pH\right)=100\left(\%\right)-\sum_{i=\text{1,2}}{p}_{i}\left(n,{pH}^{+},\sigma\:\right)+const$$

where for both p a normal density distribution

6 $$\:p\left(pH\right)={\frac{n}{\sqrt{2\pi\:\sigma}}e}^{\frac{{\left(pH-{pH}^{+}\right)}^{2}}{2{\sigma}^{2}}}$$

was chosen.

**Fig. 4:**
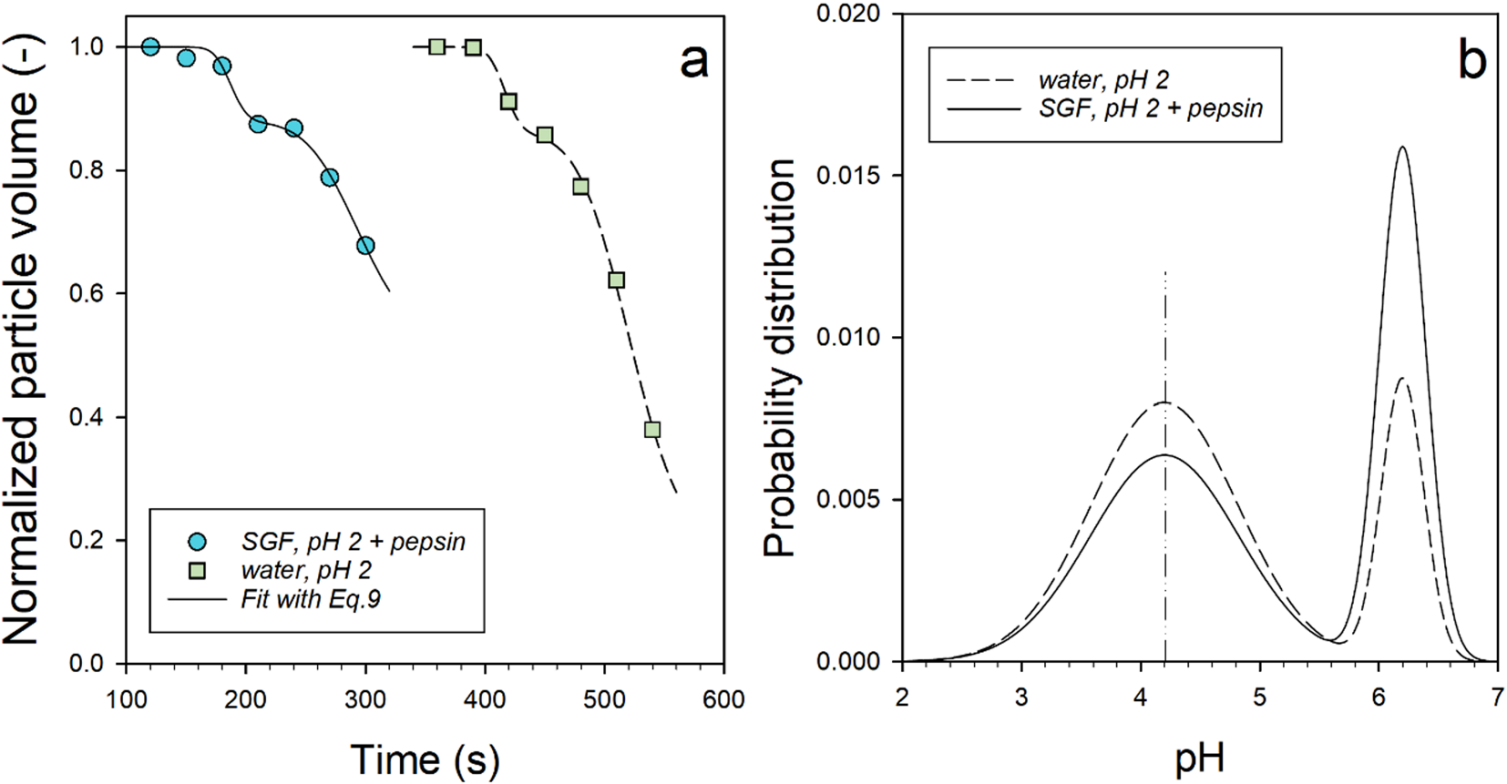
Analysis of the size change of the CMP from Figs. 2a and b in the region of the first shrinkage. (a) Decrease of the relative volume with time and fit with Eq. 9 (lines) and (b) Gaussian profiles, calculated from the values of the fit parameters plotted over the pH scale, calculated according to Eq. 10.

For a more detailed analysis of the shrinkage process of the CMPs, the particle areas of the light grey zones in Figs. 1a and b were converted into volumes using the spherical approximation and plotted in Fig. 4 normalized to the initial value of the shrinkage at t = 120 s and t = 360 s, respectively.

In the approach adopted here, the degree of insolubility of the caseins, expressed by the two Gaussian profiles, determines the strength of the solvent outflow I_V_ during the shrinkage process:

7 $$\:{I}_{V}=-V\cdot\:\sum_{i=\text{1,2}}{p}_{i}(n,{t}^{+},\sigma\:)$$

After solving the differential equation by integrating on both sides

8 $$\int_{V_{0}}^{V_{t}}\frac{dV}{V}=\int_{0}^{t}-\left({\frac{{n}_{1}}{\sqrt{2\pi\:{\sigma}_{1}}}e}^{\frac{{\left(t-{t}_{1}^{+}\right)}^{2}}{2{\sigma}_{1}^{2}}}+{\frac{{n}_{2}}{\sqrt{2\pi\:{\sigma}_{2}}}e}^{\frac{{\left(t-{t}_{2}^{+}\right)}^{2}}{2{\sigma}_{2}^{2}}}\right)dt$$

gives the following formula:

9 $$\:V\left(t\right)={V}_{0}\cdot\:{e}^{\left(-\frac{\sqrt{2\pi\:}}{2}\cdot\:{n}_{1}\cdot\:{\sigma}_{1}\cdot\:erf\left(\frac{\left(t-{t}_{1}^{+}\right)}{\sqrt{2}\cdot\:{\sigma}_{1}}\right)-\frac{\sqrt{2\pi}}{2}\cdot\:{n}_{2}\cdot\:{\sigma}_{2}\cdot\:erf\left(\frac{\left(t-{t}_{2}^{+}\right)}{\sqrt{2}\cdot\:{\sigma}_{2}}\right)\right)}$$

Equation 9 was fitted to the data in Fig. 4a using a non-linear parameter fit. For the fits, the expected values $\:{t}_{2}^{+}$ of the second error function were set equal to the times at which the minima in Figs. 2a and b (final value in phase I) were reached. Specifically, $\:{t}_{2}^{+}$ was set at 540 s for the acidification kinetics in water without pepsin and at 300 s in SGF with pepsin. We assume that the isoelectric point of the caseins (pH 4.2) is reached at these times, i.e. the pH value at which the caseins have minimum solubility and therefore the greatest volume outflow is caused for shrinkage step 2. In addition, the half-width for the first shrinkage process, σ_1_ was set to 11 s^2^ for both kinetics. The fitted values and the constraints used to analyze the data in Fig. 4a are shown in Table 1. With the values for the times $\:{t}_{1}^{+}$ and $\:{t}_{2}^{+}$ and the assumption that the pH value decreases linearly under simulated stomach conditions^19,20^, the time scale can be converted into a pH scale according to:

10 $$\:pH={pH}_{1}^{+}-\frac{\left(t-{t}_{1}^{+}\right)}{\left({t}_{2}^{+}-{t}_{1}^{+}\right)}\cdot\:\left({pH}_{1}^{+}-{pH}_{2}^{+}\right)$$

With $\:{pH}_{1}^{+}=6.2$ and $\:{pH}_{2}^{+}=4.2$ the mean pH values of the two insolubility peaks at T = 20 °C in Fig. 3 were used for this purpose. The normalised probability distributions calculated from the fitted values can then be plotted as a function of the pH scale defined by Eq. 10. They provide a model-based indication of how the insolubility of the caseins in the CMPs changes during acidification. As shown in Fig. 4b, the Gaussian profiles are very similar for both acidification kinetics.

Specifically, the position of the solubility maximum in the pH 5.4–5.8 range is not affected by pepsin, in agreement with the literature^38,39^.

**Table 1:** Values for the parameters of Eq. 9 for fitting the data in Fig. 4a. (* fixed value)

Parameters	pH 2 + pepsin	pH 2
n_1_	0.0116	0.0135
σ_1_ (s^2^)	11^*^	11^*^
$\:{t}_{1}^{+}$ (s)	187.5	416.8
n_2_	0.015	0.0426
σ_2_ (s^2^)	35.15	37.86
$\:{t}_{2}^{+}$ (s)	300^*^	540^*^

### Analysis of the swelling behaviour (Phase II)

In this study, we observe more than two swelling steps (see Fig. 2b at 3, 9 and 10 min) and that the final swelling step has a lower rate than the previous one (see Figs. 2a and b). Previous studies in acidic media at pH 0 and pH 3 showed that CMPs remained stable without size changes over a long period of time^18,41^. The more or less pronounced initial swelling step upon changing to a pH 2 medium is therefore unusual and requires further investigation.

It is unusual that the swelling process in the second, white-shaded area in Figs. 2a and b starts at a high rate and then proceeds at a lower rate. However, in previous studies conducted under alkaline conditions, a low rate swelling step was always followed by a high rate decomposition step^18^. It is also remarkable how quickly the swelling in phase II can follow the deswelling process in phase I. In fact, this behavior can also be explained by the change in the solubility of the caseins at the isoelectric point. When the isoelectric point is crossed, as described in Chap. "Analysis of the initial shrinkage process (Phase I)", the solubility of the caseins improves due to positive charging and increasing repulsion. The re-swelling of the caseins is superimposed on a one-step degradation process of the CMPs at a slower rate. As the pH value falls below pH 3 during the progressive acidification, the rate of swelling slows down as the solubility of the caseins hardly changes (see Fig. 3). Finally, the expansion process in phase II is determined only by the slow decomposition. The resulting expansion of the CMPs can be simulated using system dynamics and is described in Sect. "System dynamics modelling during pH reduction with Pepsin".

### Analysis of the pepsin-induced shrinkage process (Phase III)

As can be seen in Fig. 2a, the surface area of the particles decreases almost exponentially from the 9th minute and reaches a stable final value after approximately 30 min. As in the first two-step shrinkage, the CMPs are again compacted, but in this case the process is mainly triggered by protease activity. Exponential enzymatic in-solution degradation kinetics to a stable final value have also been observed for synthetic polymers^42^. However, the long time delay observed here until enzymatic degradation starts requires an explanation. This is provided by experiments carried out on milk samples in a human gastric simulator^20,24^. The pH value falls almost linearly to its target value of pH 2 within 1 to 2 h during the digestion of casein-containing solutions in SGF^20^. At pH 2, a maximum in the pH activity profile of pepsin was observed for the degradation of micellar casein aggregates^23^. During acidification, however, the hydrolysis of the κ−casein surface layer by pepsin already leads to the formation of clots, excluding the enzyme from the interior of the casein network formed due to the coagulation of the micelles. The structure and porosity of the clots formed is critical for pepsin diffusion into the interior of the network and the rate of casein proteolysis^24^.

**Fig. 5:**
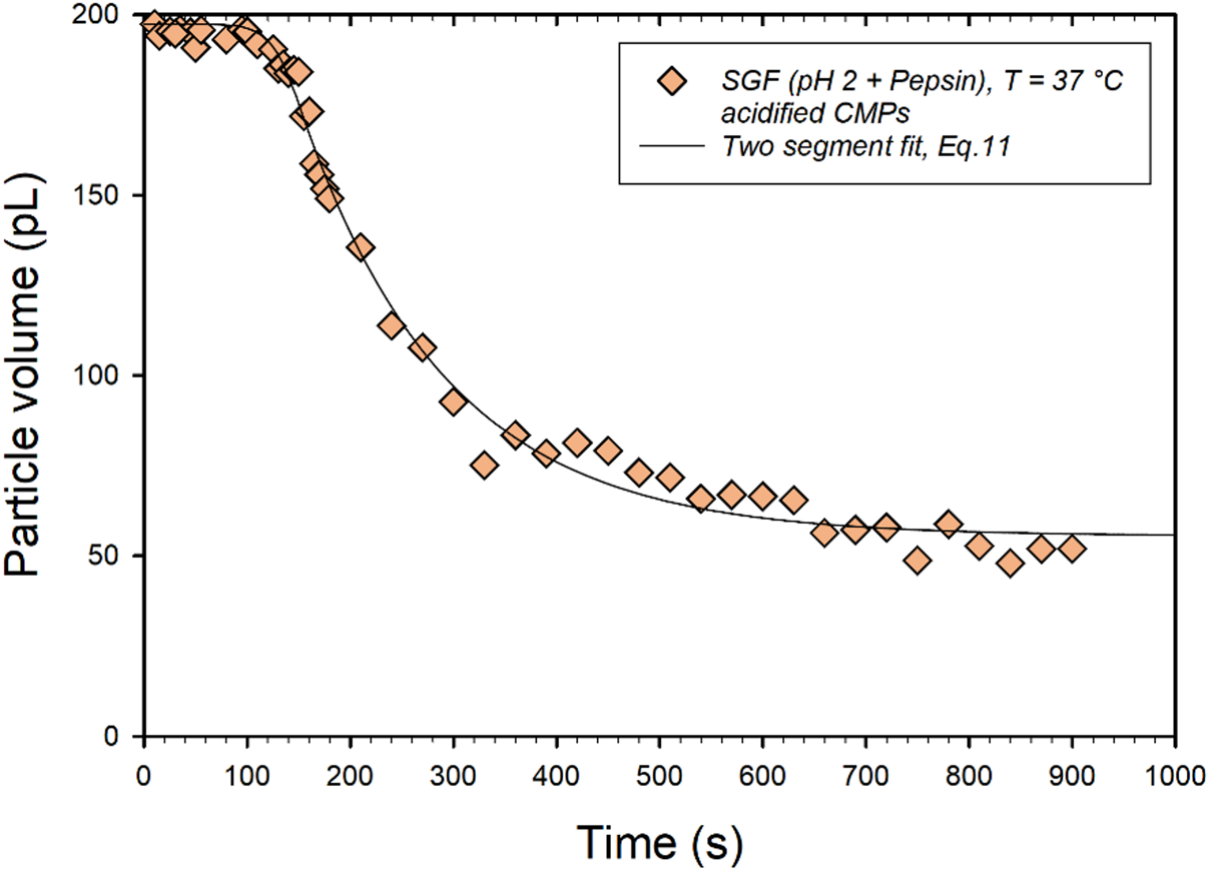
Relative volume shrinkage of CMPs previously stored in SGF without pepsin at pH 2 for 20 min after addition of 50 u pepsin/mL. Non-linear piecewise fit (solid line) with an erf for t < 150 s and with an exponential for t > 150 s, according to Eq. 11 and parameter values in Table 2.

Even before acidification, CMPs have a porous casein network structure (see Fig. 1a), which is reinforced by calcium bridges. In the swelling cell, CMPs are exposed to SGF, pH 2, under almost ideal conditions. Therefore, the acidification process is expected to be much faster than when real milk samples are digested for several hours in a human stomach simulator^20,24^. It is therefore plausible that a pH value of 2 is reached after 9 min, so that the proteolysis of total casein by pepsin can take place under optimal conditions. This assumption is supported by control experiments carried out on already acidified CMPs that were stored for 20 min in pepsin-free SGF at pH 2. Caseins are positively charged under these conditions and the stabilizing calcium bridges are dissolved^35^. The internal structure of the CMPs can therefore be considered flexible and open-pored. After a short shrinkage phase, the size of the CMPs immediately decreases exponentially to a stable final value as a result of the overflow with pepsin-containing SGF (see Fig. 2c). This happens much faster (already after 2 min) due to the porous casein-network structure, in agreement with the finding that digestion is accelerated in samples with reduced micellar calcium and more open-pored clots of heat-treated milk^24^. In order to find a suitable functional relationship for an overall system dynamics model based on volume balance, the data in Fig. 2c were converted into volumes using the spherical approximation (Fig. 5).

The error function already used in Eq. 9 proved to be the most appropriate to describe the variability of the data in the first 147s. The weak but significant transition is probably due to a contraction caused by the hydrolysis of the κ−casein on the surface of the micelles. After t > 147 s, pepsin has penetrated the inner CMPs structure and proteolysis of the total casein starts. The course of the curve can be described in good approximation by an exponential decline to a plateau value V∞. Both phases of structural change can be combined in the following functional relationship:

11 $$\:V\left(t\right)=\left\{\begin{array}{c}{V}_{0}\cdot\:{e}^{-\frac{\sqrt{2\pi\:}}{2}\cdot\:{n}_{3}\cdot\:{\sigma}_{3}\cdot\:erf\left(\frac{\left(t-{t}_{3}^{+}\right)}{\sqrt{2}\cdot\:{\sigma\:}_{3}}\right)}\:if\:t<147\:s\\{V}_{4}\cdot\:{e}^{-\left(\frac{t}{{t}_{4}}\right)}+{V}_{\infty\:}\:\:\:\:\:\:\:\:\:\:\:\:\:\:\:\:\:\:\:\:\:\:\:\:if\:t\:\ge\:147\:s\end{array}\right.$$

As shown in Fig. 5, the data points can be fitted to a good approximation with Eq. 11 using a segment fit. A rate of 7∙10^–3^ s^-1^ was determined for the exponential decay, which corresponds to a time constant of t_4_ = 142 s. A value of 56 pL was obtained for the plateau, V∞, which corresponds to 16% of the initial volume. In this state, the CMPs should be present in a very compact structure of undigested casein peptides stabilized by hydrogen bonds^25,43^.

**Table 2:** Values of the parameters in Eq. 11 for fitting the data in Fig. 5 (* fixed value).

Parameters	stored in SGF, pH 2 for 20 min before pepsin addition
V_0_ (pL)	197.4
σ_3_ (s^2^)	37^*^
$\:{t}_{3}^{+}$ (s)	193.5
V_4_ (pL)	122.3
$\:{t}_{4}$ (s)	141.7
V_∞_ (pL)	55.5

### System dynamics modelling during pH reduction with Pepsin

**Fig. 6:**
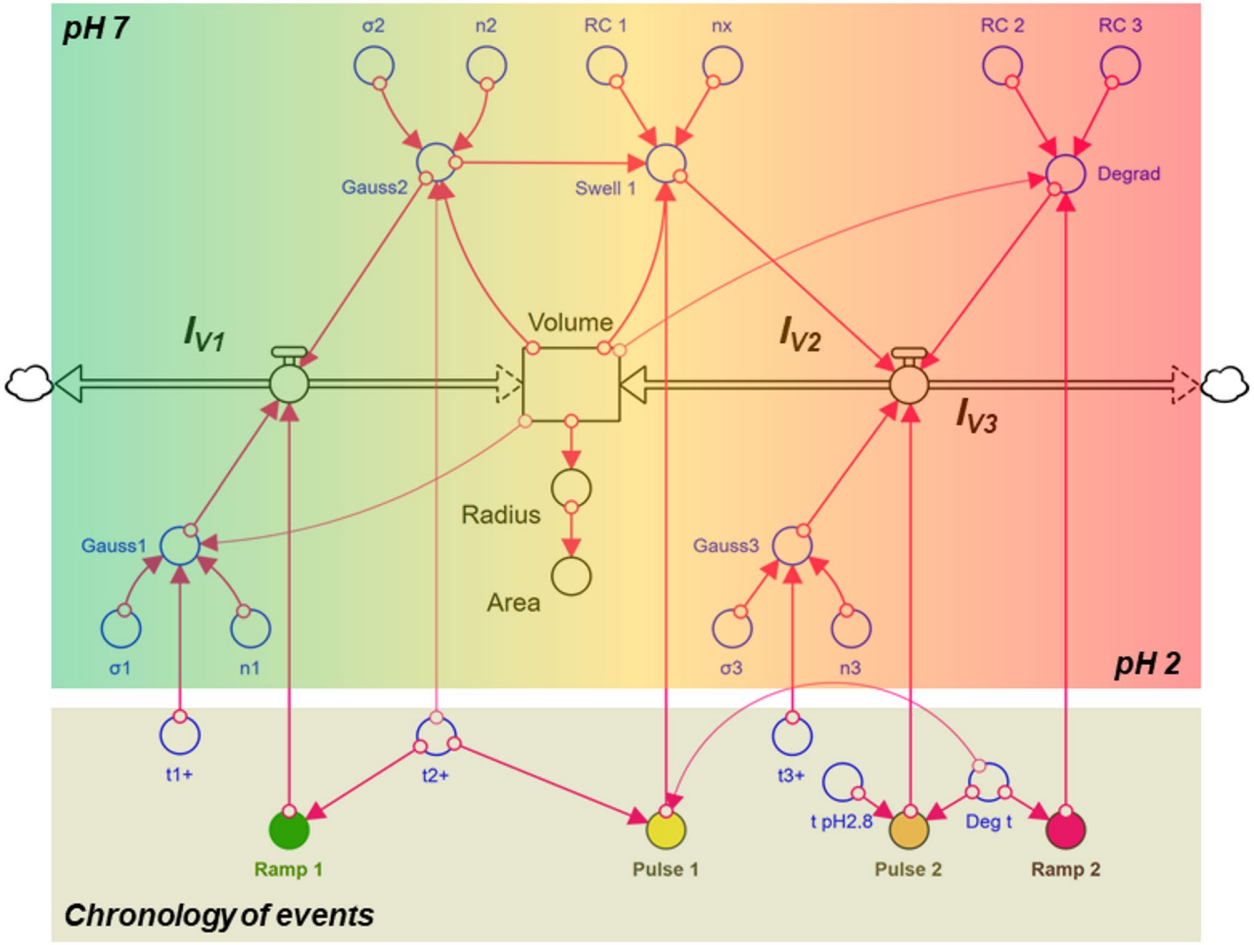
Stella model for the dynamic simulation of the complex size change observed in CMPs after replacement of the buffer by pepsin-added SGF at pH 2. The volume changes of the particle are exclusively caused by volume flows - reduction via - IV1 or - IV3, which cause two shrinkage transitions and one an exponential decay, and enlargement via IV2, which leads to a two-step expansion. The start and duration of each process (chronology of events) are determined by characteristic times that define ramps and pulses. The optically observed particle area is calculated from the volume using spherical approximation.

**Fig. 7:**
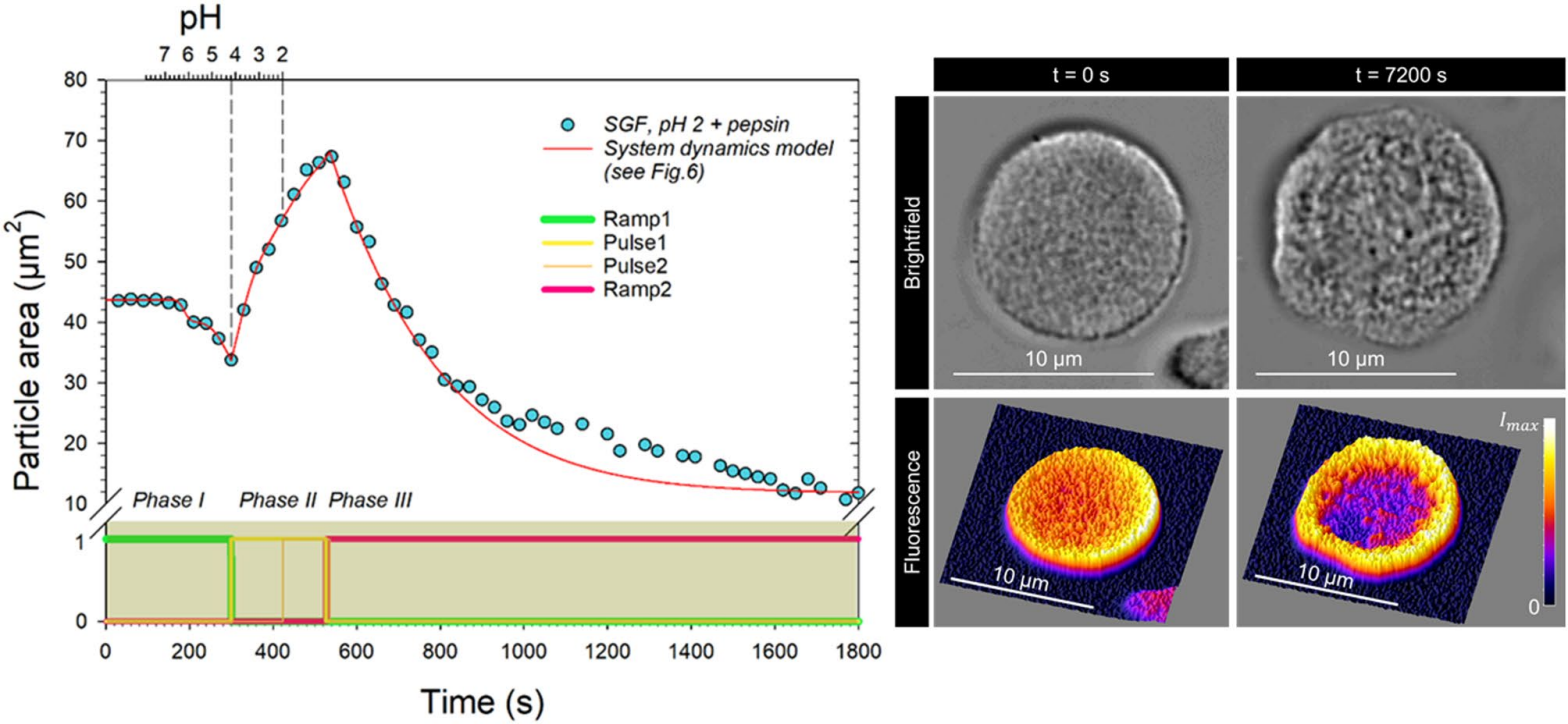
(left) System dynamics analysis of the data from Fig. 1a using the model from Fig. 6. A pH scale assuming Eq. 10 is shown at the top, the ramps and pulses used for each process are shown at the bottom; (right) Microscopic comparison of the bright−field surface structure and the confocal fluorescence resolved internal structure of two different CMPs before and after 2 h of SGF treatment.

Based on the analysis of the individual processes, an overall system dynamics model was created to simulate the data in Fig. 2a. The underlying flow chart is shown in Fig. 6 and the simulation result for the change in absolute particle area in Fig. 7. Since the spherical shape of the CMPs remained unchanged throughout the acidification and pepsin treatment, the change in particle area can be attributed to changes in volume via the spherical approximation (Eq. 4). In Eq. 1 this is triggered by three volume flows. The shrinkage in phase I and the decay in phase III are simulated by the outflows - I_V1_ and - I_V3_ and the swelling process in phase II by the volume inflow I_V2_.

12 $$\:\frac{dV}{dt}=\dot{V}=-{I}_{V1}+{I}_{V2}-{I}_{V3}$$

(12)

The time sequence of the inflows and outflows is controlled via ramps and pulses (see Fig. 6, below). The underlying formulae can be found in the programme code (see Code availability). The model has 15 parameters, whereby 5 parameter values were taken from the previous kinetic analyses in Chaps. "Analysis of the initial shrinkage process (Phase I)"–"Analysis of the pepsin-induced shrinkage process (Phase III)" and 10 parameter values (Table 3) were adapted to the data.

The initial shrinkage process (phase I) within the first 300 s was simulated by integrating the two Gaussian profiles gauss1 and gauss2 (Fig. 6). The mean values $\:{t}_{1}^{+}$ and $\:{t}_{2}^{+}$ and the half-widths σ1 and σ2 were taken from Table 1 and only n_1_ and n_2_ were re-set (see Table 3). This allowed the water loss of the inner network structure in Chap. "Analysis of the initial shrinkage process (Phase I)" to be linked to the changes in the solubility of the caseins that occur during acidification. The volume loss is greatest when the caseins are maximally insoluble and a particularly dense network is formed. The pH scale corresponding to the time scale (Eq. 10) is also shown in Fig. 7 for better characterization. The insolubility maxima of the caseins at pH 6.2 and 4.2 (Fig. 3) are passed at the points of maximum deswelling rate, i.e. at the inflection points of the two shrinkage transitions in Fig. 7 after 188 s and 300 s. The transitions are above and below pH 5 and thus in a range where clot formation starts for milk in the human stomach simulator^24^.

At the inflection point of the second shrinkage step, the particle area reaches its smallest expansion before it swells significantly. This means that at the minimum at $\:{t}_{2}^{+}$ = 300 s the solvent flow changes direction and now flows into the particle instead of out of it.

The total duration of the swelling (phase II) is determined by pulse I and lasts from $\:{t}_{2}^{+}$ = 300 s to the starting point of the exponential, enzymatic decay at Degt = 536 s. In phase II, the swelling initially occurs at a high rate, flattens significantly from t = 400 s and decreases again significantly at the peak of the swelling. As explained in Sect. "Analysis of the swelling behaviour (Phase II)", such a swelling curve is atypical, as the rate in CMPs generally increases from step to step^18^. However, the expansion observed in SGF and acidified water in Phase II (Fig. 2a and b) can be described as a simple swelling step according to Eq. 2, superimposed by two further processes. Initially, the rate of swelling I is also influenced by the high insolubility of the caseins described by gauss2. However, as shown in Fig. 4b, this decreases rapidly with decreasing pH and reaches vanishingly small values below pH 3. In contrast, effects caused by increasing pepsin activity become more significant. For CMPs stored in SGF, shrinkage was observed after the addition of pepsin (see Fig. 5), before the actual exponential degradation process started. This effect, probably due to the digestion of κ−casein on the surface of the casein micelles, could also cause shrinkage in Fig. 7, which greatly reduces the swelling rate towards the end of phase II. To model the rate in the overall model, we use gauss3, which corresponds to the description of the first shrinkage transition in Fig. 5 with the error function in Eq. 11. As indicated in the time structure of pulse 2, shrinkage starts at t(pH2.8) = 424 s, under pH conditions close to the maximum pepsin activity in the particle, and ends with the onset of exponential degradation.

Phase III is started by the ramp function Ramp2 at the time Degt = 536 s. The exponential decay to a constant value shown in Eq. 11 is described in the dynamic model by the volume outflow IV3. This corresponds to the rate coefficient RC2 multiplied by the current volume of the CMP minus the volume change rate RC3 (see Code availability). The projection area A_∞_ of the particles at the end of the kinetics then results from the ratio of the rate coefficients according to π^(1/3)^∙(3/4·RC3/RC2)^(2/3)^. With A_∞_ = 11.8 µm^2^, it has shrunk to 27% of its initial size as a result of the SGF environment and pepsin activity. In terms of particle volume, this corresponds to a shrinkage to 14% of the initial value, which is in good agreement with the value of the particle treated with pepsin only after 20 min of acidification (16%, see Fig. 5).

Although the integrity of the particles remains intact even after 2 h of SGF treatment, clear changes due to the influence of acid and protease can be detected. CMPs of similar size were selected for comparative examination by confocal fluorescence microscopy performed outside the swelling cell (see Fig. 7, right). The microstructure resolved in bright field is visibly looser and more inhomogeneous after 7200 s SGF treatment. When the distribution of fluorescence-labelled casein is examined in a section of the CMPs by confocal fluorescence microscopy, the almost complete absence of casein in the interior of the particles is striking. This strong reduction of the fluorescence signal in the centre can be attributed to pepsin activity, which causes released peptide fragments to re-aggregate at the more stable and fluorescent edge, or free fluorophore to diffuse outwards.

**Table 3:** Values of the system dynamic model parameters used to simulate the data in Fig. 7 (*fixed value).

Parameters	SGF, pH 2 + pepsin	
V_0_ (fL)	217.45	
n_1_	0.000423	
n_2_	0.000170	*Phase I*
RC1 (s^-1^)	0.0032	
nx	1.5	
n_3_	0.0141	
$\:{t}_{3}^{+}$ (s)	510	
σ_3_ (s^2^)	37.16	
t(pH 2.8) (s)	424	*Phase II*
RC2 (s^-1^)	0.005	
RC3 (fL∙s^-1^)	0.1533	
Degt (s)	536	*Phase III*

## Conclusion

The study investigated the change of CMPs in vitro under simulated gastric conditions. Microscopic in−situ single particle studies in swelling cells reveal for the first time a complex shrinkage and swelling behavior of particulate casein during acidification and enzymatic pepsin activity. Based on kinetic analyses of the data and pH-dependent solubility studies of caseins, we have developed a system dynamics model to understand and predict the size changes. The model provides a basis for coupling the release kinetics of encapsulated bioactive compounds to analyses the structure-function relationships of the microcapsules. In the future, the approach could also be extended to time-resolved size measurements of sub-micron casein carriers, which are already used as nanocapsules of polyphenols, vitamins and drugs, using X-ray, light and neutron scattering techniques. To spatially resolve the pH-dependent changes in the capsule material, swelling experiments using a pH-sensitive fluorescent dye in combination with fast confocal imaging could be used. In the future, the rheological properties of the CMPs during the shrinking and expansion process should also be taken into account. Realistic conditions for the gastric digestion process can be simulated by fluid movements, such as those induced in the stomach by muscle contraction, and by adjusting the viscosity and density of the surrounding medium.

## Methods

### Materials

Casein powder MC80 (Milei GmbH, Leutkirch im Allgäu, Germany) and highly methylated citrus pectin (CU 201, DE° > 70%) from Herbstreith & Fox GmbH & Co. KG (Neuenbürg, Germany) were in use. Sodium hydroxide (NaOH), potassium hydroxide (KOH) (> 90%), hydrochloric acid (1 M) and pectinase from Aspergillus niger were purchased from Merck KGaA (Darmstadt, Germany). All solutions were prepared with purified Milli-Q water from the water purification system used (Simplicity UV System, Merck KGaA, Darmstadt, Germany).

### Preparation of the solutions used

To prepare a 2% pectin solution, 1 g of pectin was added to 49 g of BisTris buffer solution (50 mM, pH 6.8) and allowed to dissolve completely at 80 °C for 2 h with continuous stirring. The clear solution was then adjusted to pH 6.8 with NaOH (1 M).

The simulated milk ultrafiltrate (SMUF) was prepared according to the method of Dumpler et al. 2017^44^. The salts and chemicals used were purchased from VWR LLC (Radnor, PA USA). The salts were dissolved sequentially in 500 ml Milli-Q water with stirring. The pH was adjusted to 6.7 with KOH solution. To inhibit the growth of microorganisms, sodium azide (Carl Roth GmbH + Co. KG, Karlsruhe, Germany) was also added to the solution at a concentration of 0.5 g/L.

All salts required for the preparation of simulated gastric juice (SGF) were purchased from Merck KGaA (Darmstadt, Germany). Specifically, 0.9 mM potassium chloride, 0.9 mM potassium dihydrogen phosphate, 25 mM sodium bicarbonate, 0.12 mM magnesium chloride hexahydrate, 0.5 mM ammonium carbonate and 47.2 mM sodium chloride and 0.15 mM calcium chloride hexahydrate were successively dissolved in ultrapure water at room temperature and the pH was adjusted to 2 with hydrochloric acid^45^. To investigate enzymatic digestion, pepsin (obtained from porcine gastric mucosa; Sigma-Aldrich Chemie GmbH, Schnelldorf, Germany) with appropriate activity was also added to the solution. The enzyme was previously dissolved in a solution of hydrochloric acid (pH 2) with slow stirring at 4 °C.

### Preparation of CMPs

One gram of casein powder MC80 was dissolved in nine grams of SMUF buffer by stirring at room temperature for 1 h, at 4 °C for 4 h and then at 37 °C for 1 h, each at 400 rpm. To induce the formation of casein aggregates, casein solution, pectin solution and BisTris buffer solution were mixed to obtain a solution containing 3 w% casein and 0.3 w% pectin at pH 6.8^46^. The non-adsorbing pectin is displaced from the area surrounding the casein micelles. This causes the micelles to come together due to the resulting osmotic pressure gradient, forming aggregates measuring a few micrometres in size. Petri dishes of 70 mm diameter were filled with 3.9 g of the mixture. During subsequent film drying, these aggregates solidified into stable particles^17,18^. After a drying time of 16 h, the dried films were covered with 10 ml of pectinase BisTris solution (35 u/ml) and shaken in a ThermoMixer (Eppendorf, Eppendorf AG, Hamburg, Germany) at 47 °C and 160 rpm for two hours. Hydrolysis was carried out under mild conditions so that the structure of the CMPs was not affected. During the hydrolysis reaction, the pectin matrix was degraded and the CMPs were released. The pectin hydrolysate with the free CMPs was removed from the Petri dishes and centrifuged at 22 °C, 1500 RCF, for 10 min. The liquid phase was removed and the remaining pellet was redissolved in BisTris buffer (50 mM, pH 6.8) or SGF (pH 2) and stored at 4 °C in the refrigerator until further use. The released CMPs showed high structural stability, i.e. decomposition can only be induced under extreme pH conditions or by adding SDS or citric acid^18,41^.

### Optical analysis and swelling kinetics

Swelling/deswelling kinetics were investigated on single CMPs in 3D printed flow cells with sieve hole design (Fig. 1b) fabricated using a J850Pro printer (Stratasys, MN, USA). The flow cell is connected to a PHD ULTRA™ syringe pump (Harvard Apparatus, MA, USA) via polyethylene tubing (Ø 0.5 mm), which delivers exchange medium at a flow rate of 0.07 ml per minute. The swelling chamber is first flooded with a particle dispersion and then left for a few minutes to allow individual CMPs to sediment in the sieve holes. Microscopic observation of individual CMPs was performed using a Leica DMIL LED inverted microscope (Leica Microsystems, GmbH, Wetzlar, Germany). The swelling process of the CMPs was initiated by replacing the buffer solution of the CMPs with SGF, SGF with pepsin or buffer solution with pepsin. A Basler camera (Basler AG, Ahrensburg, Germany) and Basler video recording software were used to record the structural changes in the microscope at a rate of 2 frames per second. The images were extracted using PyCharm (version 2021.1.3, JetBrains, Czech Republic) and the area of the CMPs was calculated using ImageJ software (NIH, USA) (Fig. 1c). Pepsin swelling assays were performed at 37 °C. The PE tube was passed through a heated tube (HT 63-20P-F24, Hillesheim GmbH, Waghäusel, Germany). Swelling tests in ultrapure water (pH 2) without pepsin were performed at room temperature (20 °C).

In addition, individual CMPs were analysed under constant conditions using confocal laser scanning microscopy (LSM 980 with Airyscan 2, Carl Zeiss AG, Oberkochen, Germany). CMPs were stained with the fluorophore pyranine, 8-hydroxypyrene-1,3,6-trisulfonic acid trisodium salt (Merck KGaA, Darmstadt, Germany) at a ratio of 5 mg per gram of casein. Excitation was recorded at a wavelength of 353 nm and emission at 465 nm using a 32-channel GaAsP detector at a scan rate of 11 lines per second. The images were stored at a resolution of 491 × 491 pixels and a bit depth of 16 bits per pixel.

### Kinetic analysis and system dynamics modelling

Since the CMPs retained their spherical shape during the structural changes caused by media exchange, the calculated projection areas could be converted into volumes using a spherical approximation^18^. To analyze the structural changes, rate functions were derived for parts of the kinetics and Wolfram|Alpha (2024 Wolfram Alpha LLC) was used to solve these differential equations. The resulting functions were fitted to the data using non-linear curve fitting (SigmaPlot14, Systat Software, Inc., San Jose, CA, USA). Based on the rate equations and analysis results, a system dynamics model was developed to analyze the overall kinetics of structural changes using Stella 1.6 software (iseesystems.com, Lebanon, NH). The simulation of the underlying differential equation (see supplementary note) system was performed using the Euler integration method with a simulation time step of 0.25 s.

### Statistics

After more or less pronounced initial swelling, all six CMPs analyzed showed the typical shrinkage process, followed by steep swelling and a final degradation in ultrapure water, pH 2 (see Fig. 2b).

From pepsin activities > 50 u/mL, all three CMPs previously treated with SGF, pH 2, showed an exponential decrease as shown in Fig. 2c.

The complex course of size changes shown in Fig. 2a, consisting of acid-induced deswelling and swelling and subsequent enzymatic shrinkage, were observed to varying degrees in CMPs regardless of the enzyme activity used (100, 300 and 800 u/ml) in three different sample preparations as a characteristic response to the exchange of medium with pepsin-added SGF, pH 2.

The parameter values for the simulation result in Fig. 7 were determined for the acid-induced deswelling process (t < 300 s) by non-linear fitting of the simplest model Eq. 9 to the original data (R^2^ = 0.9943). The parameter values for the subsequent swelling and enzymatic shrinkage process were optimized using the differential evolution method (implemented in the Stella model analysis tool).

## Supplementary Information

Below is the link to the electronic supplementary material.

Supplementary Material 1

## Data Availability

Own data measured for this study, all fits and simulation results have been made available at: https://doi.org/10.6084/m9.figshare.28070429.

## Code availability

The code of the STELLA simulation model to solve the underlying differential equation system has been made available under the link: 10.6084/m9.figshare.28082585.
