# Supplementary material for: Kinetic description of changes in the size of casein microparticles under simulated gastric conditions
Source: Sci Rep. 2025 Oct 10;15:35406. doi: 10.1038/s41598-025-22216-7 (PMC12514214; doi:10.1038/s41598-025-22216-7)
Supplement: Supplementary file 1 — Supplementary Material 1 [file 41598_2025_22216_MOESM1_ESM.docx]

'Kinetic Description of Changes in the Size of Casein Microparticles Under Simulated Gastric Conditions

Ronald Gebhardt* and Calvin Hohn

Chair of Soft Matter Process Engineering (AVT.SMP), RWTH Aachen University, 52074 Aachen, Germany

*Correspondence: ronald.gebhardt@avt.rwth-aachen.de; Tel.: +49 241 80 47803

R.G.: <https://orcid.org/0000-0002-9702-1996>


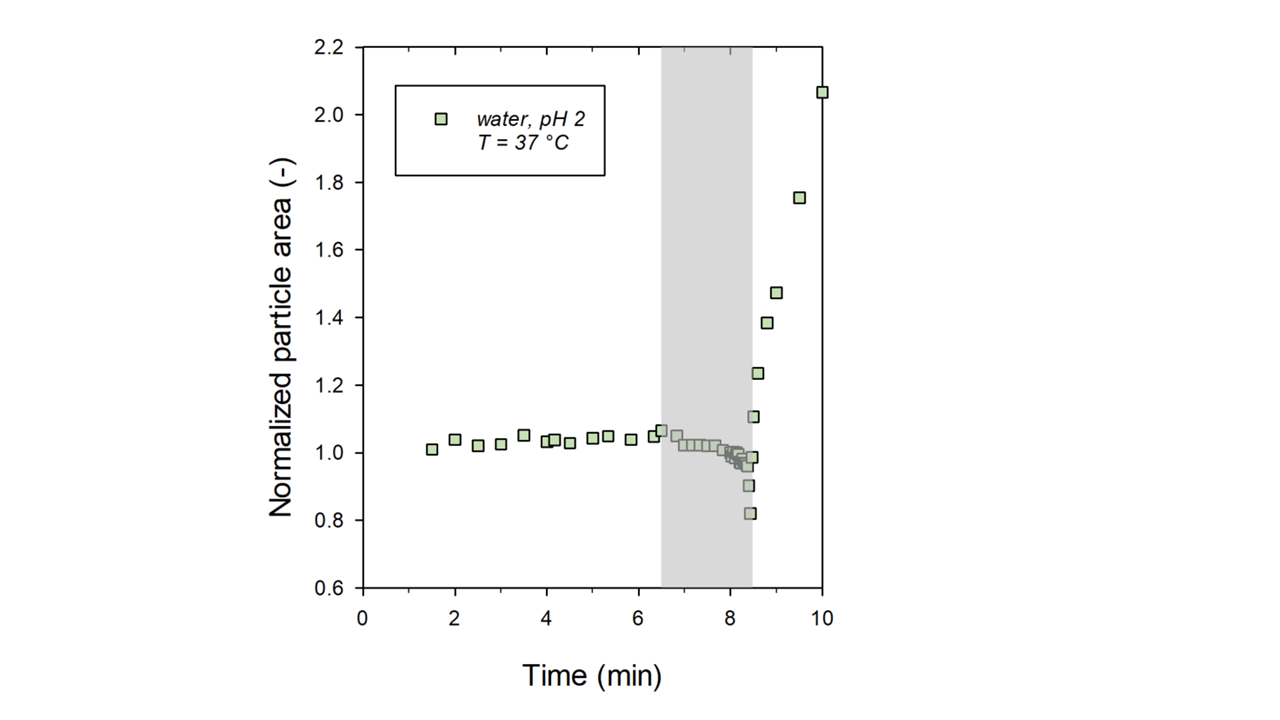


**Fig. S1:** Relative change in the size of a single representative CMPs over time following a change in pH to 2 using ultrapure water mixed with HCl at a temperature of 37 °C.
